# Operative Surgery on the Cadaver

**Published:** 1888-02

**Authors:** 


**Operative Surgery on the Cadaver.** By JASPER JEWETT GARMANY, A. M., M. D., New York: D. Appleton & Co. 1887.

This book is intended as a guide to the ordinary surgical operations. The importance of rehearsing important surgical operations on the cadaver is acknowledged by even the most eminent surgeons, and younger men should avail themselves of every opportunity of the kind offered. Whether such a book as this is necessary is, however, questionable. The surgeon who is preparing himself for an operation, ought not to need it. The advanced student can better prepare himself by the study of the subject in the great works on operative surgery.

